# Is a Nutrition Education Intervention Associated with a Higher Intake of Fruit and Vegetables and Improved Nutritional Knowledge among Housewives in Mauritius?

**DOI:** 10.3390/nu8120723

**Published:** 2016-11-29

**Authors:** Komeela Cannoosamy, Dhandevi Pem, Suress Bhagwant, Rajesh Jeewon

**Affiliations:** 1 Department of Health Sciences, Faculty of Science, University of Mauritius, Réduit, Moka 80837, Mauritius; komeela_2905@hotmail.com (K.C.); dhandevi.pem@gmail.com (D.P.); 2 Marine & Ocean Science, Fisheries & Mariculture, University of Mauritius, Réduit, Moka 80837, Mauritius

**Keywords:** nutrition knowledge, attitude, fruit and vegetable, body mass index

## Abstract

The purpose of the study was to assess the determinants of nutrition behaviors and body mass index and determine the impact of a nutrition education intervention (NEI) among Mauritian housewives. A pretest-posttest design was used assessing Nutrition Knowledge (NK), Nutrition Attitudes, Fruit and Vegetable Intake (FVI), body mass index (BMI). Two hundred Mauritian housewives were recruited. The NEI was in the form of a lecture and lasted for twenty minutes. Statistical tests performed revealed that the mean NK score at baseline was 65.8 ± 6.92 and a significant increase of +17.1 at post-test and +16.1 at follow-up was observed. Determinants of NK were age, presence of elderly people, and BMI. Mean nutrition attitude score at baseline was 2.37 ± 0.22 with significant increase of +0.2 (post-test) and +0.17 at follow-up. Age, level of education, presence of elders, and NK were linked to a positive attitude. FVI was predicted by age, income, presence of elders, NK, and nutrition attitudes. Baseline FVI was 4.77 ± 1.11 which increased significantly (*p* < 0.001) to 4.98 ± 1.13 at post-test and 5.03 ± 1.20 at follow up. NEI had a positive impact suggesting the benefits of such intervention in the promotion of healthy nutrition behaviors.

## 1. Introduction

In Mauritius, the health burden of non-communicable diseases (NCD) are increasing with a prevalence of 23.6% type II diabetes, 43.3% obesity, and 37.9% hypertension [1]. In developing countries like Mauritius, much of the rise in NCDs is attributable to modifiable risk factors such as physical inactivity and unhealthy diet which include excessive salt, fat, and sugar intake. The fact that non-communicable diseases are, to some extent, the result of individual and social patterns of behavior means that positive changes in individual dietary behavior and food- and physical activity-related policies may lead to a reduction in the risk of disease [2]. Moreover, existing evidence suggests that more than half of the NCD burden could be prevented through few key health promotions and disease prevention interventions that address such risk factors [1]. Consuming a healthy diet is considered one of the core set of preventive health strategies that is effective [3]. National and international bodies have established recommendations of what constitute a healthy diet, that is, a diet low in saturated and trans fats and high in fruits, vegetables, and grain foods. However, few adults eat in a way that consistently meets these recommendations. Clearly, there is a need to develop effective behavioral nutrition interventions that can positively and cost effectively change the dietary behavior of adults [4]. One way to combat the rise in the prevalence of nutrition-related health problems is to increase people’s nutrition knowledge, relying on the assumption that exposing an individual to new information is a necessary condition to increase nutrition knowledge. This will possibly evoke changes in attitude and subsequently resulting in improvements in dietary behavior [5]. Dietary behaviors including food choices are influenced by numerous environmental and individual factors. Some of the individual factors include socio-economic status (SES) and psychosocial factors such as knowledge, beliefs and perceptions about nutrition and health [6]. One important pathway to dietary intervention is through nutrition education which can lead to development of sufficient and balanced nutrition habits and getting rid of the bad nutrition habits. Additionally, nutrition education will act as a basic precaution for mitigating and avoiding nutrition problems that may arise due to incorrect knowledge of women on healthy dietary practices [7]. Powers et al. [8] found significant greater improvement in nutrition knowledge post the nutrition intervention in the treatment group. Likewise, significantly greater overall dietary behavior was observed in the treatment group in the contrast to the control group. Dietary behaviors are affected by many factors including nutrition knowledge. For example, individuals who have been taught about the health benefits of eating larger quantities of fruits and vegetables are more likely to consume these foods than those who were not taught [9]. Attitudes toward nutrition and health also affect dietary behavior. In Mauritius, given the increased prevalence of diseases like diabetes, obesity, cancer, and cardiovascular diseases [10], improving the dietary behaviors of the population becomes important. The entire concept of healthy nutrition behaviors may be encouraged through food and nutrition interventions. Unfortunately, in Mauritius, research on the impact of nutrition education interventions on nutrition behaviors of housewives, who are more often the main meal planner in the household [11], has not been fully explored. Consequently, this intervention study was initiated with set objectives as follows:

1. To assess the nutrition knowledge, nutrition attitudes, and fruit and vegetable intake of the housewives before and after the nutrition intervention.
2. To assess the differences in nutrition knowledge, attitudes, fruit and vegetable intake, and BMI between the determinants categories.
3. To determine the impact of the nutrition education intervention on the participants’ nutrition knowledge, attitudes, fruit and vegetable intake, and body mass index.

## 2. Materials and Methods

### 2.1. Participants

Using convenience sampling, a total of 200 housewives were recruited for the study. Inclusion criteria included being employed and voluntary participation. Women who previously attended a nutrition education intervention or were pregnant were excluded. A questionnaire consisting of five sections was used. The first section assessed nutrition knowledge where questions from Parmenter and Wardle [12] were adapted. The second section on nutrition attitudes involved attitude statements [13] with which participants had to indicate whether they agree, are uncertain, or disagree. During scoring, the direction of the question was considered such that responses of ‘Disagree’ for negatively worded statements and ‘Agree’ for positively worded statements both received a score of 3. A mean score for each participant was then calculated and could range from 3 (positive attitude) to 1 (negative attitude). The third section assessed mean fruit and vegetable intake using the Fruit and Vegetable Questionnaire (FV-Q) by Godin et al. [14]. Anthropometric measurements were recorded in the fourth section. The heights and weights of the housewives were measured to the nearest 0.1 cm and 0.1 kg respectively. Prior to each weighing, the measuring scale was adjusted to zero to enhance validity. Body mass index was calculated using weight in kilograms divided by the square of height in meters (kg/m^2^) and classified using the range of the World Health Organization [15] as shown in Table 1. The last section collected demographic data (education level, age, income, and family size).

### 2.2. Data Gathering Procedure

After obtaining informed consent, the participants were asked to fill out the questionnaire for the first time, and this served as the baseline data. Participants who were not at ease with reading and/or had difficulty writing down the answers were assisted; the questions were read and the answers were jotted down. The nutrition education intervention was in the form of a lecture and contained information on general nutrition; the food groups; portion/serving sizes; calories: meaning and importance; how to plan a healthy diet; and on fruits and vegetables, that is, the recommended intake, their importance, and benefits to health. The intervention was set to last for twenty minutes. At the end of the lecture, queries from the participants were entertained. Educational materials related to fruit and vegetable intake and benefits were provided to participants as well. Three weeks after the intervention, participants were requested to fill the questionnaire a second time (post-test). Afterwards, re-evaluation of the nutrition behaviors was instituted a third time, two months after the post-test (follow-up test).

### 2.3. Statistical Analysis

Data analysis was performed using the Statistical Package for the Social Science (SPSS)^®^ version 20.0 (IBM, Armonk, New York, NY, USA). Statistical analyses were performed using independent samples *t* test and analysis of variance to assess differences in baseline nutrition knowledge, nutrition attitudes, and fruit and vegetable intake between the different determinant categories. To assess the impact of the nutrition on the nutrition behaviors and anthropometric measurements, a paired samples *t* test was used. For each of those tests, a *p* value of less than 0.05 was considered as statistically significant.

## 3. Results

### 3.1. Demographic Characteristics

Of the 200 housewives, 40% were aged 40–49 years old, 68% had a secondary education level, 44% had an income level ranging between 644 USD and 966 USD and 76% were married. A household size of four was more common (48%) with 80% having children and 16% being a caregiver (presence of elderly person in household) (Table 2).

### 3.2. Nutrition Behaviors and Body Mass Index

As seen in Table 3, at baseline, mean knowledge score was 65.8 ± 6.92 and was associated with age (*p* < 0.001), being a caregiver (*p* < 0.001,) and body mass index (*p* = 0.044). Income (*p* = 0.169), education (*p* = 0.743), marital status (*p* = 0.992), and having children (*p* = 0.062) did not influence nutrition knowledge score. Mean nutrition attitude score at baseline was 2.37 ± 0.22. Age, higher income and education level, being a caregiver, and higher nutrition knowledge were significantly (*p* < 0.001) associated with nutrition attitude. Mean fruit and vegetable intake at baseline was 4.77 ± 1.11 servings per day. Predicting factors (*p* < 0.001) of intake were age, higher income, being a caregiver, nutrition knowledge, and positive attitude. Education (*p* = 0.207), marital status (*p* = 0.714), and having children (*p* = 0.885) did not affect fruit and vegetable intake. Mean body mass index at pre-test was 22.5 ± 3.87 kg·m^−2^. Age was found to affect body mass index such that as age increases, body mass index also increases. Similarly, a high household income and education level led to a lower body mass index value. Participants with positive nutrition attitudes, high nutrition knowledge, and high fruit and vegetable intake had lower body mass index values.

### 3.3. Impact of Nutrition Education on Nutrition Behaviors and Body Mass Index

**Figure 1:**
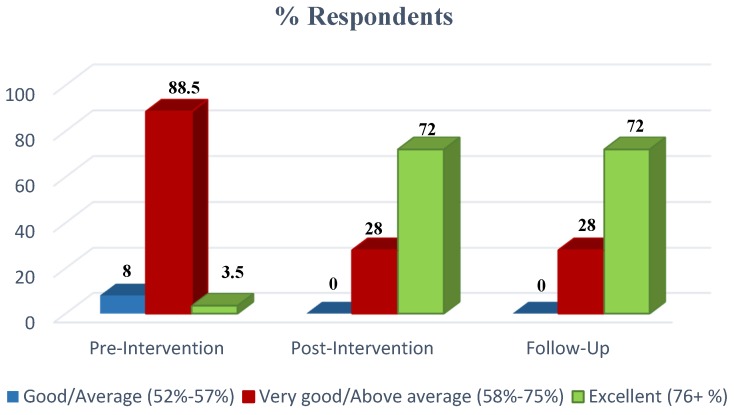
Mean nutrition knowledge score.

**Figure 2:**
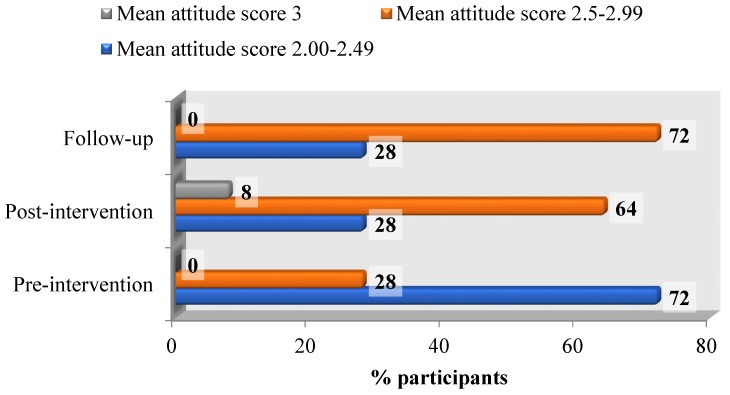
Mean attitude score.

Significant increase in the nutrition knowledge score was observed in the mean nutrition knowledge score at baseline and post-test (mean change = +17.1, *p* < 0.001). Of the participants, 92% had higher nutrition knowledge at post-test and the remaining 8% had a nutrition knowledge score similar to their baseline score. A similar significant increment in mean nutrition knowledge score between the baseline and follow-up was observed (mean change = +16.1, *p* < 0.001) (Figure 1). A significant difference was observed in the pre-test and post-test nutrition attitudes scores (mean change = +0.2, *p* < 0.001). The post-test nutrition attitudes score was higher than the pre-test score for 68% of the participants while the rest had the same pre- and post-test score. Similarly, the difference in the mean score between the pre-test and follow-up was consistent (mean change = +0.17, *p* < 0.001). A higher nutrition attitudes score was observed in the follow up test for 64% of housewives while 36% had similar scores (Figure 2). The nutrition intervention had a significant impact on the fruit and vegetable intake of housewives with an increased post-intervention fruit and vegetable intake (mean change = +0.21, *p* < 0.001). Of the participants, 12% decreased their fruit and vegetable intake post intervention, 16% had the same fruit and vegetable intake, while 72% increased their intake. During the follow up test, the mean change was +0.26 (*p* < 0.001). The fruit and vegetable intake during the follow up test decreased for 4%, remained the same for 12% and increased for 84% of the participants.

A significant difference was observed in the pre-test and post-test nutrition attitudes scores (mean change = +0.2, *p* < 0.001). The post-test nutrition attitudes score was higher than the pre-test score for 68% of the participants while the rest had the same pre- and post-test score. Similarly, the difference in the mean score between the pre-test and follow-up was consistent (mean change = +0.17, *p* < 0.001). A higher nutrition attitudes score was observed in the follow up test for 64% of housewives while 36% had similar scores (Figure 2). The nutrition intervention had a significant impact on the fruit and vegetable intake of housewives with an increased post-intervention fruit and vegetable intake (mean change = +0.21, *p* < 0.001). Of the participants, 12% decreased their fruit and vegetable intake post intervention, 16% had the same fruit and vegetable intake, while 72% increased their intake. During the follow up test, the mean change was +0.26 (*p* < 0.001). The fruit and vegetable intake during the follow up test decreased for 4%, remained the same for 12% and increased for 84% of the participants. The mean body mass index of the participants was 22.5 ± 3.87 (pre-test), 22.5 ± 3.87 (post-test) and 22.4 ± 3.85 (follow-up). The change in body mass index after the nutrition intervention was not significant (*p* = 0.751) where 10.5% of housewives had a decreased body mass index after the nutrition intervention, 10% had a greater body mass index, and the majority, that is, 79.5, had unchanged body mass index values.

## 4. Discussion

A well-planned nutrition intervention can positively impact nutrition knowledge, attitudes, and behaviors of target audiences [16]. This corroborates the major findings in this study, in particular, the positive changes in mean scores of knowledge, attitude, and fruit and vegetable intake.

### 4.1. Nutrition Knowledge

The good knowledge score obtained here may be explained by the fact that this research study included only females, and as food and health tend to be female domains [17], women are more likely to be exposed to food- and health-related information, thus gaining more knowledge. Age is positively linked to nutrition knowledge and the older age groups are known to be more health conscious and more interested in healthy eating, which in turn could result in them acquiring more nutrition knowledge [18]. Also, older respondents have higher nutrition knowledge compared to their younger counterparts as they have been exposed to more nutritional information [19]. In their study, Bhurosy and Jeewon [20] also reported higher knowledge scores in older adults after a nutrition education program. In this study, presence of an elderly person affected nutrition knowledge score whereas having children had no significant effect. According to Kim et al. [21], there are increasing numbers of families who are assuming major responsibility for taking care of aging, older adults, which may lead to an important degree of interdependence. Thus, the dietary requirements that aging brings along may increase the awareness of diet-disease associations and positively affect to importance attached to nutrition by caregivers [22,23]. However, presence of children in the household did not affect nutrition knowledge and these support findings from De Vriendt et al. [5] where having children was not a determining factor. Likewise, Williams et al. [24] found no significant correlation between maternal nutrition knowledge and children’s dietary outcomes. On the contrary, Tan et al. [25] found that the awareness rate of nutrition knowledge was higher in the parent group compared to non-parent group as parents care more about their nutrition knowledge in order to improve their children’s nutritional status. Moreover, Kakinami et al. [26] found that greater parental nutrition knowledge was associated with lower body mass index and other health related outcomes among children. Another study reported that high parental education was linked with less frequent snacking and more frequent weekly physical activity in children, compared to that of lower categories signifying the involvement of parents in nutrition intervention programs to improve dietary quality and nutritional behaviors of the entire family [27]. Body mass index (BMI) of the participants also significantly affected nutrition knowledge with participants who were underweight and obese having higher nutrition knowledge scores, 67.7 ± 9.34 and 68.2 ± 7.83, respectively. According to O’Brien and Davies [28], overweight individuals concerned about their weight may have sought out information and advice thus increasing the nutrition knowledge. Equally, underweight or normal weight individual may have an interest in their diet and sought out information on healthy eating which enhanced their nutrition knowledge scores. However, this does not always hold true as under some circumstances there is no significant relationship between nutrition knowledge and respondents’ BMI [5,29].

### 4.2. Nutrition Attitude

Younger age, household income, education level, being a caregiver, and nutrition knowledge were all significantly associated with a positive nutrition attitude. Similar results were observed by Choi et al. [30] where nutrition attitude was influenced by household income and education level. Higher nutrition attitude score was observed for higher education level and higher monthly household income. Jung [31] found that nutrition knowledge influenced nutrition attitude. According to Contento [2], people first acquire knowledge in nutrition which then changes their nutrition attitude; it is then that this change in attitude leads to dietary behavior change. Besides these influences, nutrition attitudes appear to be shaped by familial influences [32] as well as by life experiences, knowledge, and norms presented by the environment [33].

### 4.3. Fruit and Vegetable Intake

Participants aged between 30–39 years old reported the highest mean fruit and vegetable intake of 5.57 servings per day. This is in agreement with Erinosho et al. [34], who found that participants in the 35–54 age range were more likely to consume ≥5 daily servings of fruits and vegetables because adults in this age group are more likely to have children and need to provide healthy foods to the child. On the other hand, elsewhere, it was found that high fruits and vegetable consumers tend to be older [35], because as women age, they may increasingly prioritize their health for a variety of reasons, such as increased risk and awareness of certain lifestyle related diseases, which would result in higher consumption of fruits and vegetables that are characteristic of a more nutritious diet overall. In this study, having children was not found to affect fruit and vegetable consumption. The fact that having children does not increase household fruit and vegetable intake may be explained by the fact that children themselves were found to not consume adequate servings of fruits and vegetables [36]. According to Boukouvalas et al. [37], as income increases, fruit and vegetable intake also increases, however, this effect tends to level off as income gets larger. Likewise, in this study, as household income level increases, so does mean daily fruit and vegetable intake with a mean intake of 6.86 daily reported by participants with household income greater than 966 USD. Zenk et al. [38] found that women with higher household income shopped at supermarkets instead of other grocers which was associated with higher fruit and vegetable intake. Subar et al. [39] were also in accordance with our results, demonstrating a link between higher income and higher fruit and vegetable intake. Among low-income individuals, cost and transportation barriers have been cited as reasons why they do not consume fruits and vegetables more frequently [40]. Similarly, in addition to perceived availability of fruits and vegetables, financial considerations have been cited as one of the most significant barriers to healthy eating among disadvantaged women [36]. Being a caregiver was found to affect fruit and vegetable intake significantly. As aging is associated with chronic diseases, caregivers may identify fruits and vegetables as being an important part of a healthy diet [37]. The marital status of the participants did not affect the fruit and vegetable intake. Alaimo et al. [41] found that there was no difference in the fruit and vegetable intake of married and non-married participants, and eating five or more servings per day was not dependent on marital status [40]. Contradictory results were reported by Nicklett and Kadell [42] and Yannakoulia et al. [43] where marriage is positively linked to fruit and vegetable intake and married adults were most likely to achieve recommended portions and are high fruit and vegetable consumers. The benefits of marriage for fruit and vegetable intake may be due to the benefits of companionship and eating meals together [42]. A higher level of nutrition knowledge was associated with a higher intake of fruit and vegetable. Havas et al. [44] and Wolf et al. [45] reported that nutrition knowledge is associated with healthy eating, whereby knowing how many fruits and vegetables a person should eat for good health has been linked to a higher level of consumption. Also, a higher level of knowledge about the recommended daily servings of fruits and vegetables led to a 23.8% higher fruit and vegetable consumption. Grunert et al. [17], reported that higher education may facilitate learning of nutrition knowledge, but may also result in people becoming more motivated to eat healthily. This may imply a possible interrelationship between education level, nutrition knowledge, and fruit and vegetable consumption. A positive nutrition attitude (2.50–2.99) was significantly linked to a higher mean fruit and vegetable intake (6.12 servings per day). Nutrition attitudes have been reported to be relatively strong predictors of fruit and vegetable intake in adults [45] and might influence an individual’s likelihood of taking the recommended five servings of fruits and vegetable a day [46]. Also, 16% of the variance in fruit and vegetable intake may be accounted for by attitude variables [47]. Havas et al. [44] found that a positive change in attitude brought about increases in fruit and vegetable intake, and favorable attitudes can be used to predict future increases in fruits and vegetable intake [32].

### 4.4. Body Mass Index

In this study, increasing age was associated with a higher body mass index, while higher education level and household income were linked to a lower body mass index. Additionally, a higher level of nutrition knowledge, positive nutrition attitude, and a greater intake of fruits and vegetables were associated with a lower BMI. Nooyens et al. [48] reported that BMI increases with age and this increase in BMI with age may be due to the decrease in energy requirements at rest. According to Johnson et al. [49], high body mass index was less frequent among well educated people, possibly because educated individuals tend to live in environments where it is encouraged to maintain an appropriate weight. Monteiro et al. [50] reported that income level tends to be a risk factor for obesity while education may be protective as a higher level of income means a greater affordability for energy dense foods. However, in this study, a higher level of income was found to be related to a lower BMI which corroborates findings of Fokeena and Jeewon [51] and Bhurosy and Jeewon [52] where participants in high a socio-economic status (SES) group had lower BMI than those in a low SES group. Similarly, nutrition knowledge, positive nutrition attitude and greater fruit and vegetable intake were related to a lower BMI. Based on O’brien and Davies [28], nutrition knowledge and nutrition attitude are interrelated, such that a gain in knowledge will lead to a positive attitude change resulting in an improved dietary change which explained the lower body mass index. Likewise, Acheampong and Haldeman [53], reported that nutrition knowledge is associated with attitude which is, in turn, significantly associated with body mass index. Higher fruit and vegetable intake is associated with a lower BMI, this in accordance with the findings of Azagba and Sharaf [54] where a negative association between fruit and vegetable intake and body mass index was observed and suggests that increasing the intake of fruits and vegetables can be an effective dietary strategy to control weight and decreases the risk of obesity.

### 4.5. Impact of Nutrition Education on Nutrition Behaviors and Body Mass Index

Results indicated that the intervention effectively increased nutrition knowledge. Similar findings were demonstrated by Powers et al. [8] where participants in the treatment group exhibited significantly greater improvement in nutrition knowledge. Inayati et al. [55] found that nutrition knowledge was significantly increased after participating in a nutrition education program with the percentage of correct answers on nutrition knowledge being higher in the intensive nutrition education group compared to the non-intensive nutrition education group. Likewise, Kostanjevec et al. [56] observed a significant improvement in nutrition knowledge, however, only participants scoring low at baseline made the most improvement while those scoring best at baseline did not improve significantly. Dissen et al. [9] also reported that nutrition interventions should aim at increasing nutrition knowledge as higher nutrition knowledge levels may lead to more positive changes and promote healthier dietary habits. There are contradictory results where knowledge scores did not increase significantly from pre-test to post-test [57] which may be due to differences in the exposure to nutrition information, family environment, and food availability and accessibility [58]. These factors were not studied in the present study. There was a positive and significant increase of +0.2 at post-test and +0.17 at follow up in nutrition attitude score. Nutrition interventions have been found to have a positive influence on attitudes related to healthy eating and nutritional habits such that in an intervention group, attitude scores were significantly higher [33,44]. Brug et al. [59] found that when nutrition intervention is tailored, there is a significantly greater positive change in attitudes compared to the non-tailored intervention group. However, Cox et al. [32] reported that nutrition attitudes are also shaped by familial influences, thus, nutrition education should be tailed to the whole family instead of being individually tailored. The nutrition intervention was effective in increasing daily fruit and vegetable intake. The change in mean daily fruit and vegetable intake post intervention was +0.21 (*p* < 0.001) while the mean change from baseline at follow up was +0.26 (*p* < 0.001). The findings are in accordance with Pomerleau et al. [60] who reported that in primary prevention interventions, fruit and vegetable intake was increased by approximately 0.1–1.4 servings per day. Allicock et al. [61] showed that the outcome evaluation of a fruit and vegetable promotion program showed a mean increase of 0.35 servings in combined fruit and vegetable intake. There are studies which reported a higher mean change in fruit and vegetable intake following a nutrition education program. Kothe et al. [62] reported that across the entire study cohort, fruit and vegetable consumption increased by 0.83 servings/day. However, Morgan et al. [63] found no treatment effect on vegetable consumption. Recently Pem et al. [64] also reported a significant increase in fruit intake but no intervention effect for vegetable intake. The differing results among the intervention studies may be explained by the fact that fruit and vegetable intake are influenced by factors like regional differences, education, income, and knowledge, but also fruit and vegetable consumption depends on the sensory appeal, time constraints, familiarity, and availability and accessibility [65]. Gough et al. [66] reported a decrease of 1.3 kg in weight and a decrease of 0.5 kg·m^−2^ in body mass index at 3 months follow up which, however, was not sustained at 12 months’ post intervention. In this study, the nutrition education intervention was not successful at decreasing waist circumference and body mass index, but it can be effective as a weight maintenance program as the majority of the participants maintained or decreased their body mass index. Failure of this intervention study to observe changes in body mass index may be due to the short duration of the study, and, as Friedrich et al. [67] reported, isolated interventions did not result in changes of BMI which can be partially because changes in body mass do not occur in a short period of time.

## 5. Conclusions

This study brought forward interesting findings that could be used to address health problems related to dietary behaviors among Mauritian main meal planners. The main implications of this study are that nutrition knowledge, nutrition attitude, fruit and vegetable intake, and body mass index are influenced by both socio-demographic factors and psychosocial factors. Therefore, future nutrition education intervention should target populations having low nutrition knowledge and fruit and vegetable intake and barriers such as age, income, and education level should be taken into consideration when designing a nutrition intervention. Future work may involve designing new strategies on how to overcome barriers, such as affordability in terms of income level and education level, to promote healthy eating behaviors.

## Figures and Tables

**Table 1:** Classification of obesity.

Classification	BMI (kg/m^2^)
Underweight	<18.5
Normal range	18.5–24.9
Overweight	25.0–29.9
Obese	≥30.0

**Table 2:** Profile characterization of sample population.

Characteristics	*n*	%
**Age (years)**		
30–39	48	24
40–49	80	40
50–59	32	16
>60	40	20
**Education level**		
Never been to school	32	16
Primary level	32	16
Secondary level	136	68
**Household income**		
<Rs 10,000 (<322 USD)	32	16
Rs 10,000 to Rs 20,000 (322–644 USD)	64	32
>Rs 20,000 to Rs 30,000 (644–966 USD)	88	44
>Rs 30,000 (>966 USD)	16	8
**Marital status**		
Single	48	24
Married	152	76
**Household size**		
1	32	16
2	8	4
3	48	24
4	96	48
≥5	16	8
**Number of persons**		
Adults		
1	48	24
2	152	76
Children		
0	40	20
1	32	16
2	120	60
3	8	4
Elders		
0	184	92
1	16	8
**Medically certified disease**		
No	64	32
Yes	136	64

**Table 3:** Nutrition behaviors and body mass index mean scores.

	Mean Scores ± SD		
	Pre-Intervention	Post-Intervention	Follow-Up
Nutrition knowledge	65.8 ± 6.92	82.9 ± 9.32	81.9 ± 9.14
Nutrition attitude	2.37 ± 0.22	2.57 ± 0.29	2.54 ± 0.26
Fruit and vegetable intake/servings per day	4.77 ± 1.11	4.98 ± 1.13	5.03 ± 1.20
Body mass index/kg·m^−2^	22.5 ± 3.87	22.5 ± 3.87	22.4 ± 3.85
